# How the zebra got its stripes: a problem with too many solutions

**DOI:** 10.1098/rsos.140452

**Published:** 2015-01-14

**Authors:** Brenda Larison, Ryan J. Harrigan, Henri A. Thomassen, Daniel I. Rubenstein, Alec M. Chan-Golston, Elizabeth Li, Thomas B. Smith

**Affiliations:** 1 Department of Ecology and Evolutionary Biology, University of California, 610 Charles E. Young Drive South, Los Angeles, CA 90095, USA; 2 Center for Tropical Research, Institute of the Environment and Sustainability, University of California, 619 Charles E. Young Drive East, Los Angeles, CA 90095, USA; 3 Institute for Evolution and Ecology, University of Tübingen, Building E, Floor 4, Auf der Morgenstelle 28, Tübingen 72076, Germany; 4 Department of Ecology and Evolutionary Biology, Princeton University, 106A Guyot Hall, Princeton, NJ 08544, USA; 5 Department of Mathematics, University of California, 520 Portola Plaza, Math Sciences Building 6363, Los Angeles, CA 90095, USA

**Keywords:** zebra, stripes, patterning, random forest, ecological predictions, species distribution modelling

## Abstract

The adaptive significance of zebra stripes has thus far eluded understanding. Many explanations have been suggested, including social cohesion, thermoregulation, predation evasion and avoidance of biting flies. Identifying the associations between phenotypic and environmental factors is essential for testing these hypotheses and substantiating existing experimental evidence. Plains zebra striping pattern varies regionally, from heavy black and white striping over the entire body in some areas to reduced stripe coverage with thinner and lighter stripes in others. We examined how well 29 environmental variables predict the variation in stripe characteristics of plains zebra across their range in Africa. In contrast to recent findings, we found no evidence that striping may have evolved to escape predators or avoid biting flies. Instead, we found that temperature successfully predicts a substantial amount of the stripe pattern variation observed in plains zebra. As this association between striping and temperature may be indicative of multiple biological processes, we suggest that the selective agents driving zebra striping are probably multifarious and complex.

## 2. Introduction

Coloration and patterning are important adaptive characteristics in many taxa [1–3] and species from fruit flies to humans exhibit environmentally correlated gradients in pigmentation that are strongly suggestive of adaptation to local environments [4–6]. Zebra stripes are among the most striking mammalian coat patterns, yet whether they are adaptive has not been established nor have the drivers of natural selection been pinpointed. The clinal modification in striping pattern that plains zebra (*Equus quagga*) exhibit (figure 2; electronic supplementary material, figure S4) suggests that environmental factors may create selective pressures that play a role in determining stripe patterns. Within this single species, individuals run the gamut in terms of striping patterns: some have strong striping over their entire body, while others have few to no stripes on the legs, and faint shadow stripes interspersed with the primary stripes along the torso. Individuals of the extinct *quagga* subspecies from South Africa had the least amount of striping, with stripes limited to the head, neck and torso. The quagga is thought to have diverged quite recently from other plains zebra and may have undergone stripe loss relatively rapidly, possibly associated with a more open, drier environment [7]. Variation in striping patterns has also figured prominently in subspecific classifications [8,9]. Distance and large barriers such as the Zambezi River may play a role in the observed variation [8,10], but the lack of genetic structuring observed among populations that exhibit divergent stripe phenotypes [11] suggests that stripe variation may represent adaptive variation rather than simply resulting from drift in isolated populations. That the degree of striping has a genetic basis is clear from a recent heritability study conducted in captive plains zebra [12].

There are a number of adaptive hypotheses for the existence of striping in zebras [13], including predation evasion [14,15], thermoregulation [14], social cohesion [16] and avoidance of biting flies [17]. In this paper we put the predation, thermoregulation and biting fly hypotheses to a spatially explicit empirical test by modelling how variation in plains zebra stripe pattern is associated with variation in environmental variables. For example, zebra are a common, possibly preferred, prey of lion [18,19]. Computer simulations with human subjects support the notion that strong black and white patterns make it difficult for predators to capture their prey [20]. Computer simulations have also shown that such patterns influence perceived size [21], speed [22] and trajectory (much like the optical illusions created by the spinning spokes of a wheel or the rotating stripes of a barber's pole) [23]. Each of these ideas depend on a strong pattern in order to produce the corresponding effect. So if zebra stripes function as an optical illusion that reduces the success of predation efforts, we would expect to see bolder, black and white stripes in areas where encounters with lions are more likely.

Striping has been shown experimentally to prevent both glossinid (tsetse) and tabanid flies from landing on surfaces [17,24,25]. For tabanids this phenomenon has been explained as reduced polarotactic attraction caused by the interspersion of weakly polarizing white stripes between the strongly polarizing and attractive black stripes [24]. However, this explanation does not work for other biting flies, such as tsetse flies, which are not polarotactic [26]. More likely, stripes may break up the silhouette of the body against a strong background [17] or create an optical illusion that confuses flies [23]. Biting flies tend to feed low on the body, especially around legs where the zebra's skin is the thinnest [24,27,28]. Thus, we expect that stripe characteristics of the legs, or perhaps even the belly, will correlate with tsetse fly prevalence if tsetse flies have played a role in the evolution of zebra stripes.

The hypothesis that stripes help zebra thermoregulate has not been tested empirically. This hypothesis is based on the idea that black and white stripes would heat up differentially, thus causing differential airflow between black and white stripes and creating eddies of air that would have a cooling effect [14]. This mechanism should work most effectively on strong, contrasting stripes, so we would predict good coverage with bold black and white striping to occur in areas in which zebra are regularly exposed to higher temperatures.

In this paper, we make the implicit assumption that striping is adaptive as we attempt to identify potential drivers of natural selection. We make an assumption typically made in studies of the adaptive significance of traits [29], that variation in current striping patterns can be explained by current environmental conditions and that the relationship between stripes and environment has remained stable over time, although the specifics of the distributions of habitats and, consequently, stripe patterns may have changed. We conduct spatially explicit modelling of plains zebra striping using a set of environmental layers that allow us to test the hypotheses that stripe characteristics are associated with predation, biting flies or temperature. In addition to these specific hypotheses about stripe thickness and stripe saturation as they relate to predicted lion, tsetse fly and temperature distributions, we also investigate available climatic and remotely sensed variables such as precipitation, surface moisture and vegetation characteristics to investigate whether these environmental predictors help explain striping patterns.

## 3. Results

To analyse the relationship between stripe phenotype and environment, we quantified stripe characteristics at 16 sites across the plains zebra range (electronic supplementary material, figure S1). One of the 16 sites is represented by a single photograph of the extinct *quagga* subspecies, which is the only quagga specimen with precise locality data such that environmental data could be assigned to it [30]. Stripe characteristics were quantified for forelegs, hind legs, torso and belly (electronic supplementary material, figure S2), and included stripe number, thickness, length and colour saturation. As a single stripe can be described by thickness, length and colour saturation, we multiplied standardized values of these traits in order to describe the overall quality of a stripe, which we call stripe definition.

We ran a random forest model [31–35] as implemented in the package *randomForest* [36,37] in R [38] using a set of 19 predictive variables. Environmental variables successfully explained at least 30% and as much as 63% of the variance for 12 of 18 striping characteristics: foreleg stripe number, thickness, saturation and overall definition, hind leg stripe thickness and definition, torso stripe number, length, thickness, saturation and definition, and belly stripe number (table 1). The most consistently important variables were isothermality (BIO3) and mean temperature of the coldest quarter (BIO11). Maximum annual vegetation as measured by the Normalized Difference Vegetation Index (NDVIMAX) and precipitation of the wettest month (BIO13) were also important for some characteristics. Estimated tsetse fly and lion distributions, by contrast, consistently failed to predict stripe pattern variation.

**Table 1.** Random forest models, the percentage of variance they explain and their ability to predict stripe characteristics of zebra at new sites. (Models with significant predictive ability are in bold.)

		models		predictions	
body part	stripe characteristic	model	% variance explained	*r*^2^	*p*
foreleg	number	BIO3 + BIO13	44	0.01	0.33
**foreleg**	**length**	**BIO11 + NDVIMAX**	**20**	**0**.**49**	**0**.**03**
**foreleg**	**thickness**	**BIO3 + BIO11**	**42**	**0**.**66**	**0**.**009**
**foreleg**	**saturation**	**BIO11 + NDVIMAX**	**37**	**0**.**42**	**0**.**05**
**foreleg**	**definition**	**BIO3 + BIO11**	**60**	**0**.**66**	**0**.**008**
hind leg	number	BIO13 + BIO15 + MAX	22	−0.10	0.57
hind leg	length	BIO3 + BIO11	13	0.36	0.07
**hind leg**	**thickness**	**BIO3 + BIO11**	**51**	**0**.**60**	**0**.**01**
hind leg	saturation	BIO11 + NDVIMAX	20	0.37	0.06
**hind leg**	**definition**	**BIO3 + BIO11**	**63**	**0**.**60**	**0**.**001**
**torso**	**number**	**BIO3 + BIO13**	**63**	**0**.**42**	**0**.**04**
torso	length	BIO3 + BIO11 + BIO13	51	0.20	0.15
torso	thickness	BIO3 + BIO11 + TREE	37	0.24	0.12
torso	saturation	BIO11 + BIO13	37	−0.01	0.37
**torso**	**definition**	**BIO3 + BIO11 + BIO13**	**40**	**0**.**45**	**0**.**04**
belly	number	BIO11 + BIO13 + NDVIMAX	45	0.03	0.31
belly	thickness	NDVIMAX	23	−0.11	0.62
belly	saturation	BIO1 + NDVIMAX	3	−0.02	0.38

**Figure 1.**
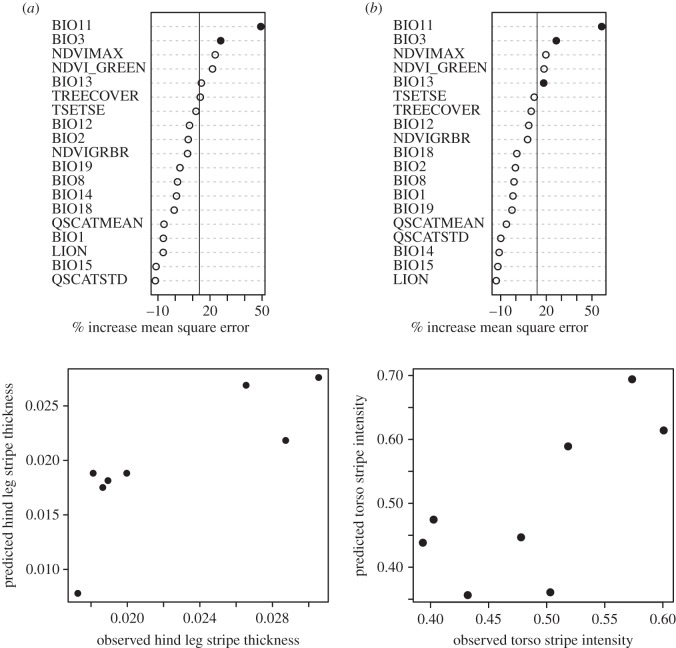
(*a*) Importance scores for each environmental variable used as input to random forest algorithm models for hind leg stripe thickness and torso stripe definition. Variables with higher mean square error (calculated as the average increase in squared residuals when the variable is permuted) are more important. Variables having an importance score greater than the absolute value of the lowest negative scoring variable (solid vertical line) are potentially important and informative [30]. Variables shown with a black circle are those that remained important as the model was refined. (*b*) Correlations between observed and predicted values for hind leg stripe thickness and torso stripe definition.

A good model has the capacity to predict characteristics for sites that were not included in the original model. We therefore tested our random forest models by quantifying stripe characteristics at eight additional sites (electronic supplementary material, figure S1) using photographs posted on the photo sharing website Flickr, or sent to us in response to requests placed there and on the University of California, Los Angeles' (UCLA) Center for Tropical Research website. We extracted the values of the best environmental predictor variables for the test sites, used these to predict the values for stripe characteristics across those sites, and regressed the predictions onto the observed values of stripe characteristics. The validity of our model estimates was well supported for stripe length, thickness, saturation and overall definition on the foreleg, thickness and definition on the hind leg, and number of stripes and definition on the torso. Our models successfully predicted the values of these eight stripe characteristics across the eight test sites (table 1). Models were also relatively successful at predicting hind leg stripe length and saturation at the test sites (table 1), while other characteristics, particularly those of the belly, were not accurately predicted for new sites. The random forest model and correlations between observed and predicted are shown in figure 1 for hind leg stripe thickness and torso stripe definition (as the quagga point is based on a single individual we reran models and predictions without that point included. Results were concordant with our previous models, electronic supplementary material, table S2).

**Figure 2.**
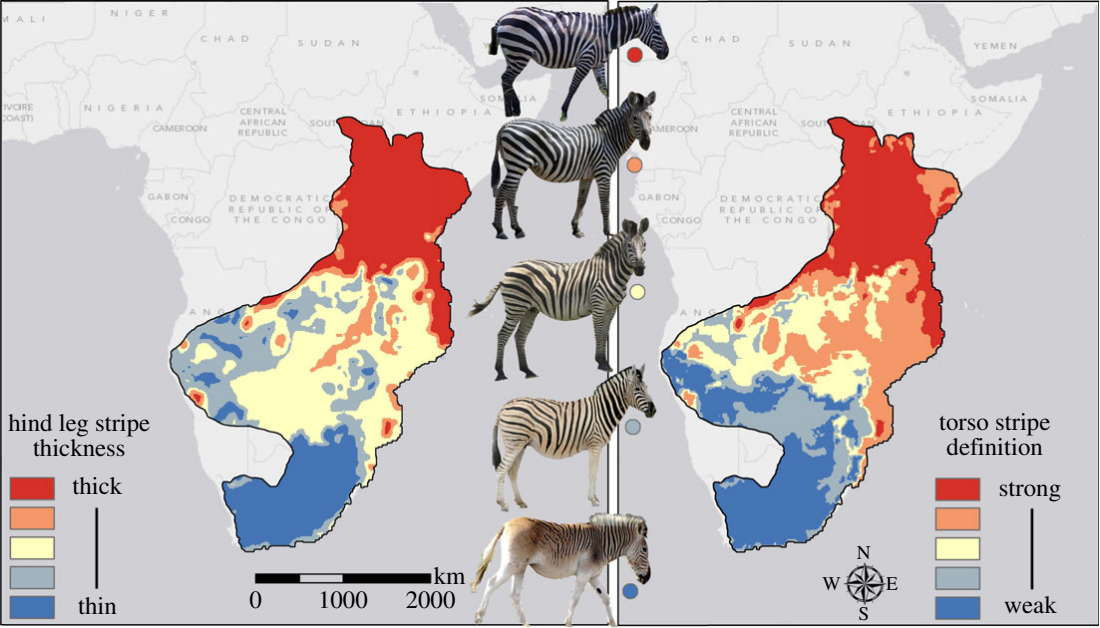
Predicted levels of hind leg stripe thickness (left) and torso stripe definition (right), from a random forest model based on 16 populations. Hind leg stripe thickness is best predicted by BIO3 and BIO11. Torso stripe definition is best predicted by BIO3, BIO11 and BIO13.

We predicted stripe characteristics across the historical plains zebra range [10] by first extracting the values of the best predictor variables for 50 000 random points across Africa using ArcGIS (Environmental Systems Research Institute, Redlands, CA). We then used a random forest model to predict values of stripe characteristics for these 50 000 points (interpolated using ordinary kriging and limited to the plains zebra known range). Figure 2 shows the predicted distribution of stripe variation for hind leg stripe thickness and torso stripe definition. Both maps show that temperature accurately predicts the general pattern of zebra striping. Interestingly, the region of Africa known to have harboured *E. quagga* and *Equus burchelli* [10], the zebra subspecies typically having no stripes on the legs, is well delineated in the map of hind leg stripe thickness, in spite of the model containing only one data point in which zebra had no leg stripes. In addition, predictions of torso stripe definition correctly identify regions at central latitudes in eastern Africa in which zebra have full length and fully saturated but thinner stripes on the torso than zebra farther north. Bivariate plots (electronic supplementary material figure S4) show the relationship between the two traits shown in figures 1 and 2 and the environmental variables identified as important by our models.

## 4. Discussion

We found that environment, particularly temperature, was a significant predictor of zebra stripe patterns across their entire range in Africa. This was supported by the large amount of variation explained by the models containing these variables, as well as the successful predictions of striping characteristics at new sites. The stripe characteristics that were most readily explained by environmental variation were stripe thickness and definition on forelegs and hind legs, and the number of stripes and definition on the torso, suggesting that these traits are the most likely targets of selection. This correlation with temperature may be explained by more than one causal mechanism, as discussed below, and will require further investigation.

Our finding that the two environmental variables most closely associated with variation in striping were both temperature variables lends support to the hypothesis that striping may be related to thermoregulation. One would expect that mechanisms to adapt to temperature would be most relevant on the torso, and the stripe characteristics of the torso are well explained by isothermality (BIO3) and mean temperature of the coldest quarter (BIO11). Given the hypothesis that stripes give rise to differential air currents that produce a cooling effect [14], intense black stripes would be expected to create more of a differential relative to white stripes, and stripe saturation is greatest in the tropics where animals experience sustained high temperatures. There have been no published direct tests of the thermoregulation hypothesis, however preliminary observations using a non-contact infrared digital thermometer gun show that zebra maintain a significantly lower surface body temperature (29.2°C vs 32.5°C) than nearby, similar sized herbivores grazing under the same conditions [39], and observations of differences in shade seeking behaviour between thin striped Grevy's zebras and thick striped plains zebras suggest stripe thickness could play a role in thermoregulation (D. I. Rubenstein 1980, personal observation).

The association between temperature and striping on the legs is not easily explained as a mechanism for thermoregulation. It may simply be a result of genetic correlation as stripe characteristics on the legs and torso are highly correlated (electronic supplementary material, table S1), or it may be a response to a different mechanism, such as avoiding fly bites. Tsetse flies and other biting flies can negatively impact animals in a number of ways: both directly, through loss of time spent foraging and energetic expenditures that lead to weight loss [40–42], and indirectly, through the transmission of disease [43]. The possibility that biting flies could be a selective agent favouring striping is supported by experimental evidence that tsetses and tabanids avoid striped surfaces [24]. However, we found no relationship between tsetse flies and variation in striping across populations, which suggests the explanation for striping in zebra is more complex than simply the avoidance of biting flies. While predicted tsetse fly distributions did not explain stripe variation, it is highly likely that the strongest selective effect of biting flies is not the bites themselves, but the diseases they may transmit. Tsetse flies, for instance, do not ubiquitously carry trypanosomes [44], partly because the ability of trypanosome infections to develop in the flies is temperature dependent, with even a small temperature difference of 3°C significantly reducing infection rates [45]. Thus, the distribution of the trypanosomes and diseases carried by tsetse flies and other flies may be quite different from the distribution of the flies themselves and could have more relevance to variation in stripe pattern. We suggest that temperature may influence trypanosome prevalence in tsetse flies and as a consequence help explain variation in striping.

A recent paper [46] compared the number of stripes on different body parts of the seven extant equid species and their subspecies. The authors found significant correlations between either tabanid or tsetse flies and various aspects of striping. However, several points should be considered when interpreting these results. Estimates of tabanid fly prevalence were not based on species distributions modelled using tabanid location data, but simply on ranges of temperature and humidity that the authors estimated to be favourable for tabanids. This both renders the reliability of the estimated tabanid ranges questionable and begs the question of whether certain ranges of temperature and/or humidity could themselves help explain observed striping patterns. The variables used represent a broad overlap between mean values of environmental variables and the species or subspecies ranges, and the use of standard statistical tests with a small number of observations meant that only variables passing individual bivariate tests for significance were added to their model. Thus, their analysis ignores the substantial variation in environmental variables that occurs within each species and subspecies range and does not allow a direct test of multiple hypotheses within the same model. Finally, their work found that the number of belly stripes in a species was positively correlated with predicted probabilities of tsetse flies. However, only one species of equid, the plains zebra, actually has both belly stripes and significant range overlap with tsetse flies, suggesting this relationship requires more rigorous analysis.

It is not particularly surprising that probabilities of lion occurrence failed to predict zebra stripe pattern, as lion are nearly ubiquitous throughout the range of plains zebra and the predicted probability of occurrence does not vary greatly (electronic supplementary material, figure S3). The predicted probability of lion in the region inhabited by the minimally striped quagga is approximately average relative to our other locations. In addition, if striping were a deterrent to lion predation, we might expect lion predation on zebra to be lower in proportion to their abundance than it is on other mammals. Instead, predation on zebra by lion has been found to be in excess of their relative abundance [18,19]. One factor that could make it difficult to detect a true effect of striping on predation using our dataset is a potential interaction between the animal pattern and the background pattern in creating camouflage or predator confusion [20,47–49]. Quantification of the backgrounds provided by the habitats inhabited by zebra would be useful.

One notable aspect of both the training and test datasets is the paucity of sites from southernmost Africa. This is due to the fact that zebra were extirpated from most of South Africa in the late 1800s resulting in the loss of the *quagga* and *burchelli* subspecies, the two with the least striping and with typically no striping on the legs. The only GIS referenced phenotype we have been able to obtain for either of these subspecies is one *quagga* specimen from the Naturhistorisches Museum Basel, which was collected from a location between Shilow and Whittlesea in South Africa [30]. The ability of this single point to help delineate the region in which zebra with no stripes on the legs used to occur is surprising and gives even greater credence to the importance of temperature in explaining the adaptive significance of striping.

Our study has shown a strong correlation between temperature and striping across the plains zebra's range. The relationship between temperature and stripe pattern is clear and, along with the lack of genetic structuring among populations [11], suggests an adaptive explanative for stripe variation. We find no clear support for the hypotheses that biting flies or predators have driven stripe evolution, however, the reduction of striping in areas with seasonally low temperatures could be a response to a reduction in the infection rate of tsetse flies with trypanosomes, a reduction in the need for convective cooling, or both. We expect the function of striping will prove to be complex given the multifarious effects of striping shown through experimentation to date, and the influence of not only temperature variables, but also the additional, albeit smaller influence, of precipitation and NDVI.

Much additional work is needed to elucidate the true functionality of striping in zebra. Our work shows a correlation with temperature, but the cause of this correlation remains unknown. More realistic experimental studies are needed to investigate whether stripes function at a distance to help zebra evade detection, and whether they function at close range to create optical illusions resulting in sufficient confusion to decrease the likelihood of being bitten or predated. For instance, experimental evidence is mixed as to whether the visual signal of striping may be overcome by the attractiveness of odours such as *CO*_2_ and ammonia [17,50] that tsetse flies use to locate animals, and direct evidence from live animals in the field is completely lacking. Although zebra blood is not typically found in surveys of tsetse fly blood meals [51,52] and screenings of wild mammals rarely identify zebra with trypanosomes [53], the same is true for many mammal species that lack stripes. Furthermore, trypanosomiasis has been found in zebra [54]. Therefore, one cannot reasonably conclude that the lack of parasite data in zebra is related to the characteristic of striping. While stripes clearly create confusion in the constrained environment of a computer screen, this same phenomenon may not occur on a larger scale under normal conditions. Larger scale experiments using live animals or a virtual system are clearly warranted. Experiments also need to be conducted to investigate the potential role of temperature. Whether stripes function in thermoregulation, how environmental factors influence tsetse trypanosome loads and likelihood of infection if bitten, all need to be studied. Finally, additional modelling, incorporating genetic data, additional sites, and new variables suggested by experimental data, should be run in order to clarify the pattern of variation and its causes.

## 5. Experimental procedures

### 5.1 Phenotype datasets

A minimum of eight zebra per site was measured for the training dataset (with the exception of the single data point for the quagga), and five or more for the test dataset. Zebra were photographed in profile with the entire body and legs (to fetlock) visible. The bulk of the photographs were taken by the authors and a few were donated by trusted individuals (other researchers or professional tour guides). As a convention, we treated the dark regions of the pelage as stripes. For each zebra, four stripe characteristics were quantified on each leg (L) and the torso (T), while three were quantified on the belly (B) (electronic supplementary material, figure S2). We quantified the number of stripes (LTB), length of each stripe (LT only, horizontally across the leg and vertically across the torso), stripe thickness (LTB) and stripe saturation (LTB). Stripe thickness was measured in pixels down the midline of the legs and torso and along the belly, and standardized by dividing by the pixel length of the body part. Stripe thickness and body part lengths were measured using ImageJ software [55]. For each stripe, however small or faint, length was estimated categorically as less than or equal to 25, 50, 75 or 100%, and saturation was estimated in categories from 0 to 7, with 0 being no stripe and 7 being black. All measurements were performed by one of three researchers. At least two zebra at each site were measured by a minimum of two researchers in order to estimate repeatability, and two photos at different exposures were taken of a subset of animals to determine the effect of exposure on estimation of colour saturation. Finally, we examined the possibility that allometry might be a concern using data on skull lengths from [8]. We found no significant correlations between stripe characteristics and exposure or size. Additional measurement details and repeatability results can be found in the electronic supplementary material.

### 5.2 Environmental variables

Variables input into random forest models included 19 temperature and precipitation variables from WorldClim [56], (http://www.worldclim.org/), and five remote-sensing variables quantifying the concentration of green leaf vegetation (MODIS-derived NDVI [57]), which are typically considered a measure of greenness [58] and productivity [59] (see electronic supplementary material, table S3). We also used the MODIS-derived vegetation continuous field (VCF) product as a measure of the percentage of tree canopy cover [60]. Monthly composites (mean and standard deviation) of global Quick Scatterometer (QSCAT) [61] are indicative of surface moisture, leaf water content and other seasonal attributes. We used the Food and Agriculture Organization's Programme Against African Trypanosomiasis data on predicted probability of tsetse fly occurrence [62]. The genus *Glossina* (tsetse flies) is divided into three groups, which comprise several species each. The Morsitans group is the most common tsetse group predicted to co-occur with plains zebra and is often the only one predicted to occur at a site, but the other two groups, Palpalis and Fuscus, are also predicted to be present in parts of the range. As multiple species of tsetse flies are capable of carrying all three species of trypanosomes that infect animals [63,64], we used a composite of the three groups as our predictor variable. We modelled predicted probabilities for lion presence using MAXENT [65] (electronic supplementary material, figure S3). Lion presence data for 116 locations was culled from various sources including the American Museum of Natural History (http://www.amnh.org/our-research/vertebrate-zoology/mammalogy/database), the National Museum of Natural History (http://vertebrates.si.edu/mammals/mammals_databases.html), the literature [19,66–75] and one personal communication (Kimura D, ranger, Lake Mburo National Park, 1980). The tsetse fly and lion data are both predicted probabilities of occurrence based on habitat suitability; we assume that zebras are more likely to encounter and be impacted by tsetse flies and lions in those regions where the probability of occurrence of these species are highest. Reduction from 29 to 19 variables was accomplished by removing all but one of each set of covarying predictors (correlation of greater than 0.9).

Zebra are very mobile and likely to experience a range of conditions within their area of movement thus variables derived from 1 km resolution layers were deemed to be too fine grained. We therefore averaged the value of each variable across a 25 km radius around the central point of the protected area within which they were photographed.

### 5.3 Random forest modelling

We ran 20 000 trees within each random forest run, removing the least important variables after each run until we identified the ‘best’ model (‘best’ was defined as the model with the highest out of bag, variance explained). See the electronic supplementary material for an explanation of random forest analysis.

## Supplementary Material

Supplementary Text, Figures and Tables

## Data accessibility

Climate and remote sensing data, MAXENT input data, and zebra phenotype data are available at http://dx.doi.org/10.5061/dryad.s13rt.

## References

[1] Caro T 2005 The adaptive significance of coloration in mammals. *BioScience* 55, 125–136. (doi:10.1641/0006-3568(2005)055[0125:TASOCI]2.0.CO;2)

[2] Endler J, Basolo A 1998 Sensory ecology, receiver biases and sexual selection. *Trends Ecol. Evol.* 13, 415–420. (doi:10.1016/S0169-5347(98)01471-2) 21238370 10.1016/s0169-5347(98)01471-2

[3] Nachman M 2005 The genetic basis of adaptation: lessons from concealing coloration in pocket mice. *Genetica* 123, 125–136. (doi:10.1007/s10709-004-2723-y) 15881685 10.1007/s10709-004-2723-y

[4] Pool JE, Aquadro CF 2007 The genetic basis of adaptive pigmentation variation in? *Drosophila melanogaster.* 16, 2844–2851. (doi:10.1111/j.1365-294X.2007.03324.x) 10.1111/j.1365-294X.2007.03324.x PMC2650379 17614900

[5] Hoekstra H, Drumm K, Nachman M 2004 Ecological genetics of adaptive color polymorphism in pocket mice: geographic variation in selected and neutral genes. *Evolution* 58, 1329–1341. (doi:10.1111/j.0014-3820.2004.tb01711.x) 15266981 10.1111/j.0014-3820.2004.tb01711.x

[6] Jablonski NG, Chaplin G 2010 Colloquium Paper: Human skin pigmentation as an adaptation to UV radiation. *Proc. Natl Acad. Sci. USA* 107, 8962–8968. (doi:10.1073/pnas.0914628107) 20445093 10.1073/pnas.0914628107 PMC3024016

[7] Leonard JA, Rohland N, Glaberman S, Fleischer RC, Caccone A, Hofreiter M 2005 A rapid loss of stripes: the evolutionary history of the extinct quagga. *Biol. Lett.* 1, 291–295. (doi:10.1098/rsbl.2005.0323) 17148190 10.1098/rsbl.2005.0323 PMC1617154

[8] Groves CP, Bell CH 2004 New investigations on the taxonomy of the zebras genus? *Equus* 69, 182–196. (doi:10.1078/1616-5047-00133)

[9] Cabrera A 1936 Subspecific and individual variation in the Burchell zebras. *J. Mammal.* 17, 89–112. (doi:10.2307/1374181)

[10] Hack MA, East R, Rubenstein DI 2002 Status and action plan for the plains zebra (Equus burchelli). *Equids* 4, 43–57.

[11] Lorenzen ED, Arctander P, Siegismund HR 2008 High variation and very low differentiation in wide ranging plains zebra (Equus quagga): insights from mtDNA and microsatellites. *Mol. Ecol.* 17, 2812–2824. (doi:10.1111/j.1365-294X.2008.03781.x) 18466230 10.1111/j.1365-294X.2008.03781.x

[12] Parsons R, Aldous-Mycock C, Perrin MR 2007 A genetic index for stripe-pattern reduction in the zebra: the quagga project. *S. Afr. J. Wildl. Res.* 37, 105–116. (doi:10.3957/0379-4369-37.2.105)

[13] Ruxton G 2002 The possible fitness benefits of striped coat coloration for zebra. *Mamm. Rev.* 32, 237–244. (doi:10.1046/j.1365-2907.2002.00108.x)

[14] Morris D 1990 *Animal watching: a field guide to animal behaviour.* London, UK: Johnathan Cape.

[15] Cloudsley-Thompson LJ 1984 How the zebra got his stripes: new solutions to an old problem. *Biologist* 31, 226–228.

[16] Kingdon J 1984 The zebra's stripes: an aid to group cohesion. In *The encyclopedia of mammals* (ed. MacDonald D). pp. 486–487. Oxford, UK: Equinox.

[17] Waage JK 1981 How the zebra got its stripes: biting flies as selective agents in the evolution of zebra coloration. *J. Entomol. Soc. S. Afr.* 44, 351–358.

[18] Hayward MW, Kerley GIH 2005 Prey preferences of the lion (Panthera leo). *J. Zool.* 267, 309–322. (doi:10.1017/S0952836905007508)

[19] Rubenstein DI 2010 *Ecology, social behavior, and conservation in zebras* 1st edn Amsterdam, The Netherlands: Elsevier Inc.

[20] Stevens M, Yule DH, Ruxton GD 2008 Dazzle coloration and prey movement. *Proc. R. Soc. B* 275, 2639–2643. (doi:10.1098/rspb.2008.0877) 10.1098/rspb.2008.0877 PMC2605810 18700203

[21] Taya S, Miura K 2007 Shrinkage in the apparent size of cylindrical objects. *Perception* 36, 3–16. (doi:10.1068/p5597) 17357702 10.1068/p5597

[22] Scott-Samuel NE, Baddeley R, Palmer CE, Cuthill IC 2011 Dazzle camouflage affects speed perception. *PLoS ONE* 6, 20233 (doi:10.1371/journal.pone.0020233) 10.1371/journal.pone.0020233 PMC3105982 21673797

[23] How MJ, Zanker JM 2014 Motion camouflage induced by zebra stripes. *Zoology* 117, 163–170. (doi:10.1016/j.zool.2013.10.004) 24368147 10.1016/j.zool.2013.10.004

[24] Egri A, Blaho M, Kriska G, Farkas R, Gyurkovszky M, Akesson S, Horvath G 2012 Polarotactic tabanids find striped patterns with brightness and/or polarization modulation least attractive: an advantage of zebra stripes. *J. Exp. Biol.* 215, 736–745. (doi:10.1242/jeb.065540) 22323196 10.1242/jeb.065540

[25] Gibson G 1992 Do tsetse flies ‘see’ zebras? A field study of the visual response of tsetse to striped targets. *Physiol. Entomol.* 17, 141–147. (doi:10.1111/j.1365-3032.1992.tb01191.x)

[26] Green CH, Cosens D 1983 Spectral responses of the tsetse fly, *Glossina morsitans morsitans.* 29, 795–800. (doi:10.1016/0022-1910(83)90009-4)

[27] Allan SA, Day JF, Edman JD 1987 Visual ecology of biting flies. *Annu. Rev. Entomol.* 32, 297–316. (doi:10.1146/annurev.en.32.010187.001501) 2880551 10.1146/annurev.en.32.010187.001501

[28] Torr SJ, Hargrove JW 1998 Factors affecting the landing and feeding responses of the tsetse fly Glossina pallidipes to a stationary ox. *Med. Vet. Entomol.* 12, 196–207. (doi:10.1046/j.1365-2915.1998.00105.x) 9622375 10.1046/j.1365-2915.1998.00105.x

[29] Weber MG, Agrawal AA 2012 Phylogeny, ecology, and the coupling of comparative and experimental approaches. *Trends Ecol. Evol.* 27, 394–403. (doi:10.1016/j.tree.2012.04.010) 22658878 10.1016/j.tree.2012.04.010

[30] Rau RE 1974 Revised list of the preserved material of the extinct Cape Colony quagga Equus quagga quagga. *Ann. S. Afr. Mus.* 65, 41–87.

[31] Prasad AM, Iverson LR, Liaw A 2006 Newer classification and regression tree techniques: bagging and random forests for ecological prediction. *Ecosystems* 9, 181–199. (doi:10.1007/s10021-005-0054-1)

[32] Strobl C, Malley J, Tutz G 2009 An introduction to recursive partitioning: rationale, application, and characteristics of classification and regression trees, bagging, and random forests. *Psychol. Methods* 14, 323–348. (doi:10.1037/a0016973) 19968396 10.1037/a0016973 PMC2927982

[33] Evans JS, Murphy MA, Holden ZA, Cushman SA 2010 Modeling species distribution and change using random forest. In *Predictive species and habitat modeling in landscape ecology* (eds Drew CA, Wiersma YF, Huettmann F), pp. 139–159. New York, NY: Springer.

[34] Svetnik V, Liaw A, Tong C, Wang T 2004 Application of Breiman's random forest to modeling structure-activity relationships of pharmaceutical molecules. In *Lecture notes in computer science* (eds Roli F, Kittler J, Windeatt T), pp. 334–343. Berlin, Germany: Springer.

[35] Larison B, Njabo KY, Chasar A, Fuller T, Harrigan RJ, Smith TB 2014 Spillover of pH1N1 to swine in Cameroon: an investigation of risk factors. *BMC Vet. Res.* 10, 1–8. (doi:10.1186/1746-6148-10-55) 24593895 10.1186/1746-6148-10-55 PMC4016523

[36] Breiman L 2001 Statistical modeling: the two cultures (with comments and a rejoinder by the author). *Stat. Sci.* 16, 199–231. (doi:10.1214/ss/1009213726)

[37] Liaw A, Wiener M 2002 Classification and regression by randomForest. *R News* 2, 18–22.

[38] R Core Team. R: a language and environment for statistical computing. Vienna, Austria: R Foundation for Statistical Computing See http://www.R-project.org/.

[39] Irondo D, Rubenstein DI In preparation Zebra stripes: their role in modulating biting flies loads and body temperature.

[40] Toupin B, Huot J, Manseau M 1996 Effect of insect harassment on the behaviour of the rivière george caribou. *Arctic* 49, 375–382. (doi:10.14430/arctic1213)

[41] Weladji RB, Almøy T 2006 Use of climatic data to assess the effect of insect harassment on the autumn weight of reindeer (Rangifer tarandus) calves. *J. Zool.* 260, 79–85. (doi:10.1017/S0952836903003510)

[42] Witter LA, Johnson CJ, Croft B, Gunn A, Gillingham MP 2011 Behavioural trade-offs in response to external stimuli: time allocation of an Arctic ungulate during varying intensities of harassment by parasitic flies. *J. Anim. Ecol.* 81, 284–295. (doi:10.1111/j.1365-2656.2011.01905.x) 21950373 10.1111/j.1365-2656.2011.01905.x

[43] Maudlin I, Holmes PH, Miles MA 2004 *The trypanosomiases* 1st edn Wallingford, UK: CABI Publishing.

[44] Jordan AM 2002 Tsetse flies as vectors of trypanosomes. *Vet. Parasitol.* 2, 143–152. (doi:10.1016/0304-4017(76)90059-5)

[45] Welburn SC, Maudlin I 1991 Rickettsia-like organisms, puparial temperature and susceptibility to trypanosome infection in? *Glossina morsitans.* 102, 201–206. (doi:10.1017/S0031182000062491) 10.1017/s0031182000062491 1852487

[46] Caro T, Izzo A, Reiner RC, Walker H, Stankowich T 2014 The function of zebra stripes. *Nat. Commun.* 5, 3535 (doi:10.1038/ncomms4535) 24691390 10.1038/ncomms4535

[47] Godfrey D, Lythgoe JN, Rumball DA 1987 Zebra stripes and tiger stripes: the spatial frequency distribution of the pattern compared to that of the background is significant in display and crypsis. *Biol. J. Linn. Soc.* 32, 427–433. (doi:10.1111/j.1095-8312.1987.tb00442.x)

[48] Anstis S 2003 Moving objects appear to slow down at low contrasts. *Neural Netw.* 16, 933–938. (doi:10.1016/S0893-6080(03)00111-4) 12850053 10.1016/S0893-6080(03)00111-4

[49] Blakemore MR, Snowden RJ 2000 Textured backgrounds alter perceived speed. *Vis. Res.* 40, 629–638. (doi:10.1016/S0042-6989(99)00214-X) 10824266 10.1016/s0042-6989(99)00214-x

[50] Blaho M, Egri A, Száz D, Kriska G, Åkesson S, Horvath G 2013 Physiology & behavior. *Physiol. Behav.* 119, 168–174. (doi:10.1016/j.physbeh.2013.06.013) 23810990 10.1016/j.physbeh.2013.06.013

[51] Muturi CN, Ouma JO, Malele II, Ngure RM, Rutto JJ, Mithöfer KM, Enyaru J, Masiga DK 2011 Tracking the feeding patterns of tsetse flies (Glossina genus) by analysis of bloodmeals using mitochondrial cytochromes genes. *PLoS ONE* 6, e17284 (doi:10.1371/journal.pone.0017284.t001) 21386971 10.1371/journal.pone.0017284 PMC3046180

[52] Clausen PH, Adeyemi I, Bauer B, Breloeer M, Salchow F, Staak C 1998 Host preferences of tsetse (Diptera: Glossinidae) based on bloodmeal identifications. *Med. Vet. Entomol.* 12, 169–180. (doi:10.1046/j.1365-2915.1998.00097.x) 9622371 10.1046/j.1365-2915.1998.00097.x

[53] Anderson NE, Mubanga J, Fevre EM, Picozzi K, Eisler MC, Thomas R, Welburn SC 2011 Characterisation of the wildlife reservoir community for human and animal trypanosomiasis in the Luangwa Valley, Zambia. *PLoS Negl. Trop. Dis.* 5, e1211 (doi:10.1371/journal.pntd.0001211.t006) 21713019 10.1371/journal.pntd.0001211 PMC3119639

[54] Mulla A, Rickman L 1988 The isolation of human serum-resistant Trypanosoma (Trypanozoon) species from zebra and impala in Luangwa Valley, Zambia. *Trans. R. Soc. Trop. Med. Hyg.* 82, 718 (doi:10.1016/0035-9203(88)90211-8) 3252589 10.1016/0035-9203(88)90211-8

[55] Schneider CA, Rasband WS, Eliceiri KW 2012 NIH Image to ImageJ: 25 years of image analysis. *Nat. Methods* 9, 671–675. (doi:10.1038/nmeth.2089) 22930834 10.1038/nmeth.2089 PMC5554542

[56] Hijmans R, Cameron S, Parra J, Jones P, Jarvis A 2005 Very high resolution interpolated climate surfaces for global land areas. *Int. J. Climatol.* 25, 1965–1978. (doi:10.1002/joc.1276)

[57] Myneni RB et al 2002 Global products of vegetation leaf area and fraction absorbed PAR from year one of MODIS data. *Remote Sens. Environ.* 83, 214–231. (doi:10.1016/S0034-4257(02)00074-3)

[58] Buermann W, Saatchi S, Smith TB, Zutta BR, Chaves JA, Mila B, Graham CH 2008 Predicting species distributions across the Amazonian and Andean regions using remote sensing data. *J. Biogeogr.* 35, 1160–1176. (doi:10.1111/j.1365-2699.2007.01858.x)

[59] Tucker CJ, Sellers PJ 1986 Satellite remote sensing of primary production. *Int. J. Remote Sens.* 7, 1395–1416. (doi:10.1080/01431168608948944)

[60] Hansen M, DeFries R, Townshend J, Sohlberg R, Dimiceli C, Carroll M 2002 Towards an operational MODIS continuous field of percent tree cover algorithm: examples using AVHRR and MODIS data. *Remote Sens. Environ.* 83, 303–319. (doi:10.1016/S0034-4257(02)00079-2)

[61] Long DG, Drinkwater MR, Holt B, Saatchi S, Bertoia C 2001 Global ice and land climate studies using scatterometer image data. *EOS Trans. Am. Geophys. Union* 82, 503 (doi:10.1029/01EO00303)

[62] Wint W, Rodgers D 2000 Consultants report: predicted distributions of tsetse in Africa, pp. 1–62. Rome, Italy: Food and Agriculture Organization of the United Nations.

[63] Rotureau B 2013 Through the dark continent: African trypanosome development in the tsetse fly. *Front Cell Infect Microbiol.* 3, 53 (doi:10.3389/fcimb.2013.00053) 24066283 10.3389/fcimb.2013.00053 PMC3776139

[64] Wilson SG, Morris K, Lewis J, Krog E 1963 The effects of typanosomiasis on rural economy: with special reference to the Sudan, Bechuanaland and West Africa. *Bull. WHO* 28, 595–613. 14001093 PMC2554950

[65] Phillips SJ, Dudik M 2008 Modeling of species distributions with MAXENT: new extensions and a comprehensive evaluation. *Ecography* 31, 161–175. (doi:10.1111/j.0906-7590.2008.5203.x)

[66] Nowell K, Jackson P 1996 *Wild cats* pp. 1–421. Gland, Switzerland: International Union for Conservation of Nature.

[67] Chardonnet P 2002 *Conservation of the African lion: contribution to a status survey.* Paris, France: International Foundation for the Conservation of Wildlife.

[68] Bauer H, Van Der Merwe S 2004 Inventory of free-ranging lions Panthera leo in Africa. *Oryx* 38, 26–31. (doi:10.1017/S0030605304000055)

[69] Haas SK, Hayssen V, Krausman PR 2005 *Panthera leo. Mamm. Spec.* 762, 1–11.

[70] Barnett R, Yamaguchi N, Barnes I, Cooper A 2006 The origin, current diversity and future conservation of the modern lion (Panthera leo). *Proc. R. Soc. B* 273, 2119–2125. (doi:10.1098/rspb.2004.2813) 10.1098/rspb.2006.3555 PMC1635511 16901830

[71] Barnett R, Yamaguchi N, Shapiro B, Nijman V 2007 Using ancient DNA techniques to identify the origin of unprovenanced museum specimens, as illustrated by the identification of a 19th century lion from Amsterdam. *Contrib. Zool.* 76, 87–94.

[72] Jacobson AP, Cattau ME, Riggio JS, Petracca LS, Fedak DA 2013 Distribution and abundance of lions in northwest Tete Province, Mozambique. *Trop. Conserv. Sci.* 6, 87–107.

[73] Riggio JS 2011 The African lion (Panthera leo leo): a continent-wide species distribution study and population analysis. Masters thesis, Duke University, Durham, NC, USA See http://hdl.handle.net/10161/3714.

[74] Celesia GG, Peterson AT, Peterhans JCK, Gnoske TP 2010 Climate and landscape correlates of African lion (Panthera leo) demography. *Afr. J. Ecol.* 48, 58–71. (doi:10.1111/j.1365-2028.2009.01082.x)

[75] Tumenta PN, Kok JS, van Rijssel JC, Buij R, Croes BM, Funston PJ, de Iongh HH, de Haes HAU 2009 Threat of rapid extermination of the lion (Panthera leo leo) in Waza National Park, Northern Cameroon. *Afr. J. Ecol.* 48, 1–7.

