# Supplementary material for: How the zebra got its stripes: a problem with too many solutions
Source: R Soc Open Sci. 2015 Jan 14;2(1):140452. doi: 10.1098/rsos.140452 (PMC4448797; doi:10.1098/rsos.140452)
Supplement: Supplementary Text, Figures and Tables [file rsos140452supp1.docx]

**How the Zebra got its stripes: A problem with too many solutions – Electronic Supplementary Material**

B. Larison, R.J. Harrigan, H.A. Thomassen, D.I. Rubenstein, A. Chan-Golston, E. Li, and T.B. Smith

**Figure S1. Sampling sites.** Model training sites are shown in black, with the quagga site represented as a star. Test sites are shown in white.

**
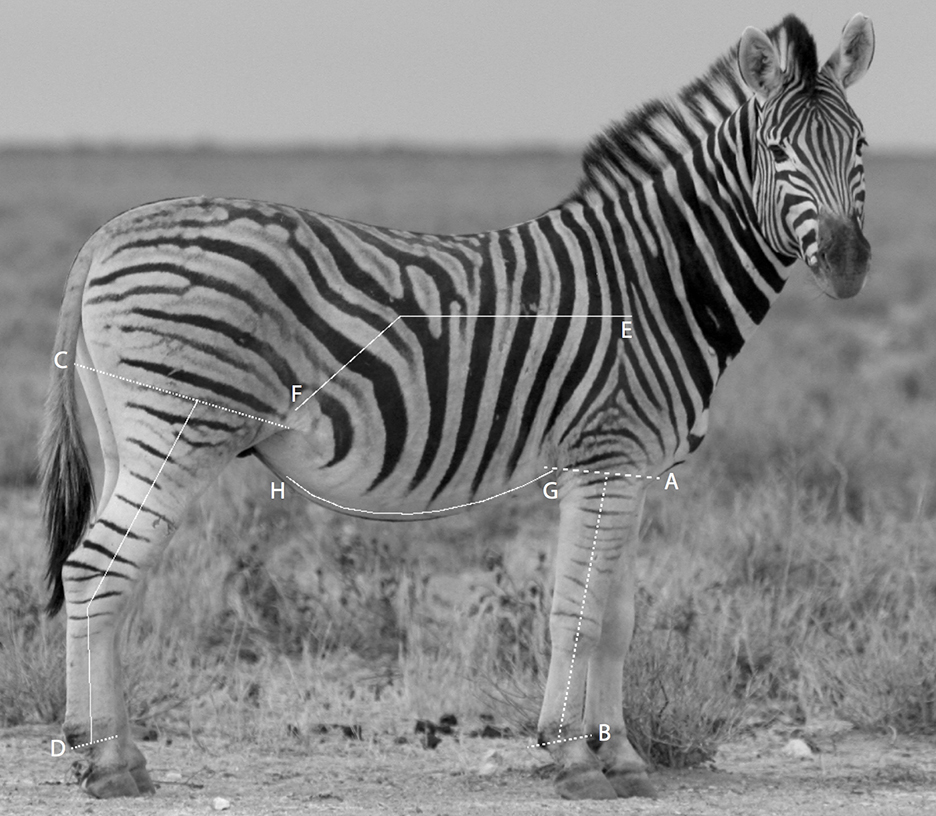
**

**Figure S2. Body part delineation and measurement of stripe characteristics.** Stripe characteristics were measured along the solid white lines. Stripes not crossing the midline were not counted. Body parts were delineated by the dashed lines as follows: foreleg – between the elbow (just below the shoulder muscle, A) and the front fetlock (B), hind leg - between line C (drawn from the groin to halfway between the dock and the back of the knee, and the rear fetlock (D), torso - from the shoulder stripe (top of the chevron over the shoulder muscle, E) to the groin (F), belly – the underside from just behind the shoulder (G) to just below the groin (H). ICC values for repeated measurements were as follows, given as mean ICC (lower bound, upperbound): stripe number: 0.94 (0.92, 0.96), stripe thickness 0.95 (0.92, 0.97, stripe length 0.98 (0.98, 0.99), stripe color Saturation 0.74 (0.64, 0.80). For measurements of color saturation of stripes in the same zebra across a one-stop exposure difference ICC was 0.89 (0.76, 0.96).


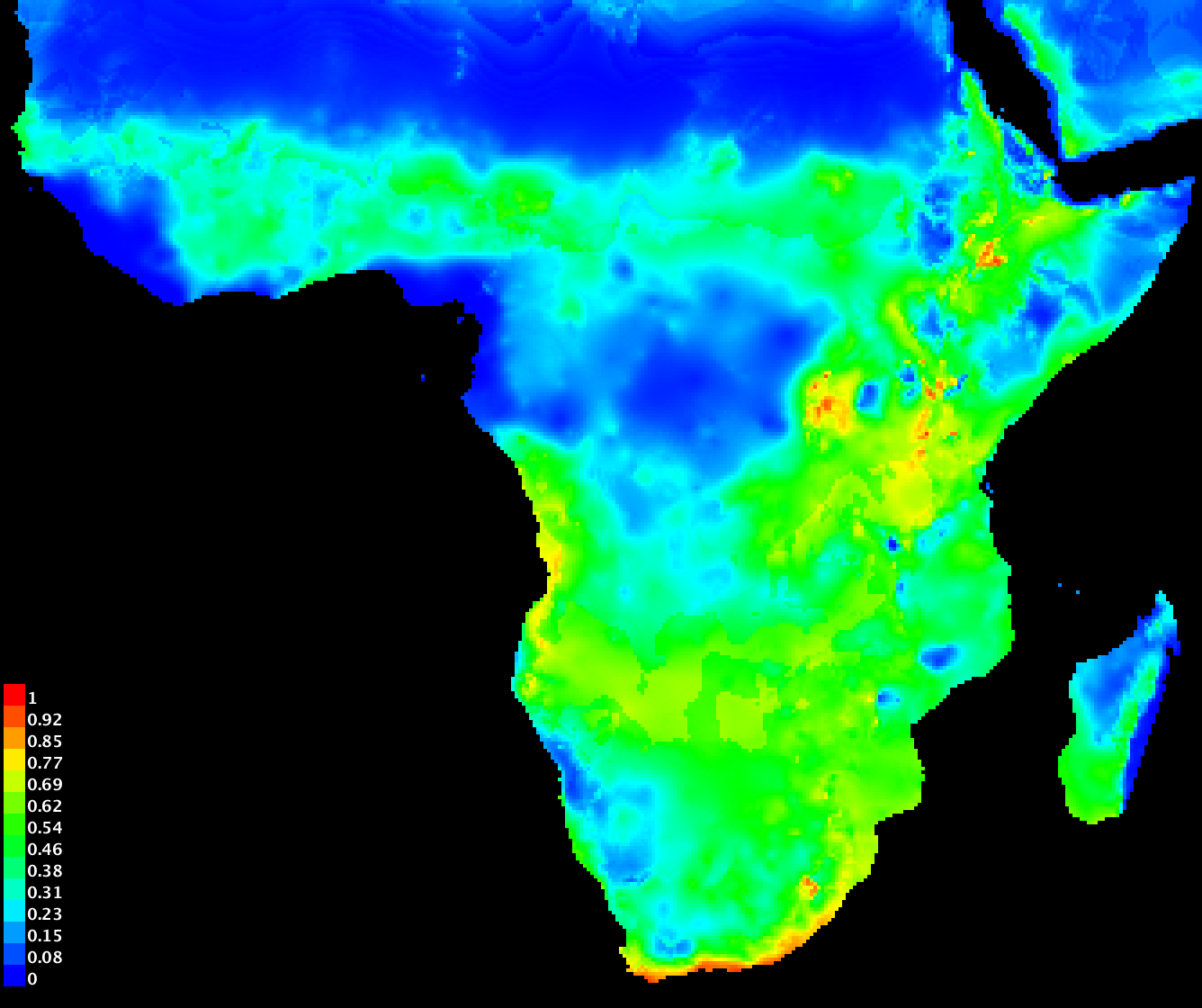


**Figure S3. Predicted probability of lions across Africa.** The predicted distribution aligns well with our understanding of historical distributions [1].

**a)**

**b)**

**Figure S4. Bivariate plots for a) Hind leg stripe thickness and b) Torso stripe definition.** A multiple regression was also run for both traits on the environmental variables identified as important by random forest: a) adjusted R-squared = 0.6918

F = 26.81 df = 21,2, *P =* *1.653e-06* b) adjusted R-squared = 0.669

F = 16.5 df = 20,3, *P = 1.239e-05.* P values for the individual variables are indicated on the plots, top value all sites, bottom value without quagga.

| **Supplemental Tables**  **Table S1. Correlations among stripe characteristics.** Bottom matrix contains correlation coefficients, top matrix contains bonferroni corrected significance. Stripe characteristics were generally correlated with each other, with the exception that most belly stripe characteristics were not correlated with each other or with the same characteristics of legs and torso. | | | | | | | | | | | | | | | | |
| --- | --- | --- | --- | --- | --- | --- | --- | --- | --- | --- | --- | --- | --- | --- | --- | --- |
|  |  | **Foreleg** | | | | **Hind leg** | | | | **Torso** | | | | **Belly** | | |
|  |  | **Number** | **Length** | **Thickness** | **Intensity** | **Number** | **Length** | **Thickness** | **Intensity** | **Number** | **Length** | **Thickness** | **Intensity** | **Number** | **Thickness** | **Intensity** |
| **Foreleg** | **Number** |  | ** | ns | ** | *** | ** | ns | ** | ns | ** | ns | ** | ns | ns | ns |
|  | **Length** | 0.86 |  | ** | **** | ns | **** | *** | **** | * | **** | *** | ** | ns | * | ns |
|  | **Thickness** | 0.51 | 0.83 |  | ** | ns | ** | **** | ** | * | * | ** | ns | ns | * | ns |
|  | **Intensity** | 0.86 | 0.98 | 0.84 |  | ns | **** | ** | *** | ** | **** | *** | *** | ns | ns | ns |
| **Hind leg** | **Number** | 0.91 | 0.71 | 0.36 | 0.72 |  | ns | ns | ns | ns | ns | ns | ns | ns | ns | ns |
|  | **Length** | 0.85 | 0.94 | 0.85 | 0.95 | 0.73 |  | *** | **** | * | **** | *** | *** | ns | * | ns |
|  | **Thickness** | 0.67 | 0.89 | 0.95 | 0.91 | 0.44 | 0.9 |  | *** | ** | **** | *** | ** | ns | * | ns |
|  | **Intensity** | 0.87 | 0.97 | 0.83 | 0.98 | 0.73 | 0.97 | 0.9 |  | ** | **** | *** | *** | ns | ns | ns |
| **Torso** | **Number** | -0.7 | -0.83 | -0.79 | -0.83 | -0.45 | -0.8 | -0.9 | -0.83 |  | ** | *** | *** | ns | ns | ns |
|  | **Length** | 0.83 | 0.93 | 0.82 | 0.94 | 0.63 | 0.92 | 0.92 | 0.94 | -0.88 |  | **** | **** | ns | ns | ns |
|  | **Thickness** | 0.71 | 0.91 | 0.88 | 0.89 | 0.54 | 0.88 | 0.91 | 0.88 | -0.9 | 0.92 |  | *** | ns | * | ns |
|  | **Intensity** | 0.84 | 0.88 | 0.75 | 0.91 | 0.66 | 0.89 | 0.86 | 0.92 | -0.9 | 0.96 | 0.89 |  | * | ns | ns |
| **Belly** | **Number** | 0.71 | 0.72 | 0.51 | 0.78 | 0.66 | 0.68 | 0.56 | 0.73 | -0.5 | 0.75 | 0.59 | 0.79 |  | ns | ns |
|  | **Thickness** | 0.57 | 0.83 | 0.8 | 0.77 | 0.4 | 0.79 | 0.8 | 0.76 | -0.62 | 0.75 | 0.81 | 0.59 | 0.37 |  | * |
|  | **Intensity** | 0.67 | 0.67 | 0.45 | 0.59 | 0.65 | 0.7 | 0.48 | 0.62 | -0.32 | 0.57 | 0.58 | 0.45 | 0.35 | 0.8 |  |
| significance levels: *<0.05, **<0.01, ***<0.001, ****<0.0001 | | | | | | |  |  |  |  |  |  |  |  |  |  |

**Table S2. Random forest models run on extant zebra only*, the percentage of variance they explain and their ability to predict stripe characteristics of zebra at new sites. Models that both explained a large amount of stripe variation and successfully predicted stripe characteristics at new sites are in bold.**

|  |  | **MODELS** | | **PREDICTIONS** | |
| --- | --- | --- | --- | --- | --- |
| **Body Part** | **Stripe Characteristic** | **Model** | **% Variance Explained** | **R2** | **P** |
| Foreleg | Number | BIO3 | 52 | 0.07 | 0.26 |
| **Foreleg** | **Length** | **BIO3** | **66** | **0.65** | **0.009** |
| **Foreleg** | **Thickness** | **BIO3+BIO11** | **62** | **0.77** | **0.002** |
| **Foreleg** | **Saturation** | **BIO3** | **57** | **0.48** | **0.03** |
| **Foreleg** | **Intensity** | **BIO3+BIO11** | **63** | **0.71** | **0.005** |
| Hind leg | Number | BIO3 | 49 | -0.14 | 0.70 |
| **Hind leg** | **Length** | **BIO3+BIO11** | **56** | **0.46** | **0.04** |
| **Hind leg** | **Thickness** | **BIO3+BIO11** | **68** | **0.77** | **0.002** |
| **Hind leg** | **Saturation** | **BIO3** | **62** | **0.40** | **0.05** |
| **Hind leg** | **Intensity** | **BIO3+BIO11** | **63** | **0.70** | **0.006** |
| **Torso** | **Number** | **BIO3+BIO11** | **60** | **0.54** | **0.02** |
| Torso | Length | BIO11 | 52 | 0.09 | 0.25 |
| Torso | Thickness | BIO3+BIO11 | 39 | 0.30 | 0.09 |
| Torso | Saturation | BIO11 | 58 | 0.23 | 0.13 |
| **Torso** | **Intensity** | **BIO3 + BIO11** | **42** | **0.40** | **0.05** |
| Belly | Number | BIO1+BIO12+BIO13 | 40 | 0.02 | 0.33 |
| Belly | Thickness | BIO3 | 21 | 0.02 | 0.33 |
| Belly | Saturation | BIO14+NDVIMAX | 15 | 0.14 | 0.20 |

*A consequence of having the quagga data point in the model is overall lower predictability of striping. This is due to the fact that the quagga is substantially different from extant zebras in terms of striping, and our data only contains a single observation for the quagga as compared to 15 for more stripy extant zebra. Thus during the crossvalidation that occurs during the random forest iterations the extreme quagga data point is often absent from the training data, driving down the variation explained by the model. However, the broad conclusions remain the same whether or not the quagga is in the model, and the predictive maps prove to be more accurate with the quagga included.

**Table S3 Predictive variables**

|  | **Variable** | **Source** |
| --- | --- | --- |
| *BIO1* | *Annual Mean Temperature* | *WorldClim* |
| *BIO2* | *Mean Diurnal Temperature Range (Mean of monthly(max-min))* | *WorldClim* |
| *BIO3* | *Isothermality – (BIO2/BIO7)*100* | *WorldClim* |
| BIO4 | Temperature Seasonality (standard deviation*100) | WorldClim |
| BIO5 | Maximum Temperature of Warmest Month | WorldClim |
| BIO6 | Minimum Temperature of Coldest Month | WorldClim |
| BIO7 | Temperature Annual Range (BIO5-BIO6) | WorldClim |
| *BIO8* | *Mean Temperature of Wettest Quarter* | *WorldClim* |
| BIO9 | Mean Temperature of Driest Quarter | WorldClim |
| BIO10 | Mean Temperature of Warmest Quarter | WorldClim |
| *BIO11* | *Mean Temperature of Coldest Quarter* | *WorldClim* |
| *BIO12* | *Annual Precipitation* | *WorldClim* |
| *BIO13* | *Precipitation of Wettest Month* | *WorldClim* |
| *BIO14* | *Precipitation of Driest Month* | *WorldClim* |
| *BIO15* | *Precipitation Seasonality (coefficient of variation)* | *WorldClim* |
| BIO16 | Precipitation of Wettest Quarter | WorldClim |
| BIO17 | Precipitation of Driest Quarter | WorldClim |
| *BIO18* | *Precipitation of Warmest Quarter* | *WorldClim* |
| *BIO19* | *Precipitation of Coldest Quarter* | *WorldClim* |
| NDVIBR | NDVI of Brownest Quarter | MODIS/NASA |
| *NDVIGR* | *NDVI of Greenest Quarter* | *MODIS/NASA* |
| *NDVIGRBR* | *NDVI Difference Between Greenest and Brownest Quarter* | *MODIS/NASA* |
| *NDVIMAX* | *Maximum Annual NDVI* | *MODIS/NASA* |
| NDVIMEAN | Mean Annual NDVI | MODIS/NASA |
| *TREE* | *Tree Canopy Cover* | *MODIS/NASA* |
| *QSCATMEAN* | *Mean Surface Moisture* | *QSCAT/NASA* |
| *QSCATSTD* | *Standard Deviation of Surface Moisture* | *QSCAT/NASA* |
| *LION* | *Predicted Probability of Lion* | *Larison/Maxent Model* |
| *TSETSE* | *Combined Predicted Probability of Glossinid Groups Morsitans, Fuscus and Palpalis* | *PAAT/FAO* |

*Variables shown in italics are the ones ultimately used in random forest analyses*

**Predicted Probabilities of Lion Occurrence**

Probabilities of lion occurrence were predicted for each site based on a species distribution model produced using MAXENT [2]. MAXENT estimates the relationship between presence data and environmental variables at known presence sites (see main text for how known presence sites were obtained), and uses this relationship to project probabilities of occurrence across the entire landscape [3]. A standard set of non-covarying WorldClim [4], <http://www.worldclim.org/>) variables, (BIO1, BIO2, BIO4, BIO5, BIO6, BIO12, BIO15, BIO16, BIO17) was used to model the predictions. As for variables used in the rest of the paper, we derived 25km averages centered on the known localities and used these to run the models. Multiple MAXENT models were run using various feature sets (H, HQ, L, LQ, T, LPQ, QTH, PQTH). We ran 10 repetitions each time, using a random 25% of the dataset as test data. We selected the model having the greatest area under the ROC curve, and for properties of the map such as no or little probability in known absence sites such as the sahara and rainforest regions. The model with hinge and quadratic features performed the best on these counts (AUC = 0.772). Further variable reduction did not improve the fit of the model so all variables were used to predict probabilities. Based on jackknife tests of variable importance, the most important variables predicting lion distributions were BIO1, BIO5, BIO12, and BIO16. The resulting map (Figure S3) fits well with the accepted historic distribution of lion on the African continent [1].

**Explanation of the Random Forest Algorithm**

Unlike standard statistical models, random forest models make no *a priori* assumptions about the relationship between the response and predictor variables, can make accurate predictions from ‘wide’ datasets containing more predictors than observations, and are less susceptible to spatial autocorrelation [5-7]. The basis of the model is a decision tree, which uses a binary recursive partitioning procedure to measure the amount of variation in a response variable explained by each predictor variable in the model, splitting the response variable successively by the variable explaining the majority of the remaining variance. Random forests use a randomized bootstrapping method in which each tree is constructed using a randomly selected subset of both the observations and predictor variables. This bootstrapping provides an intrinsic cross-validation which reduces the likelihood of over-fitting relative to more standard models [5,8,9]. The error rate of the model is based on the combined error rate of all the bootstrap iterations when the data not included in that iteration is predicted using the tree from the current iteration. Variables are ranked in order of importance as determined by the reduction in predictive accuracy of the model when that variable is permuted randomly.

**Supplementary References**

1. Barnett R, Yamaguchi N, Barnes I, Cooper A (2006) The origin, current diversity and future conservation of the modern lion (*Panthera leo*). Proceedings: Biological Sciences 273: 2119–2125. doi:10.1098/rspb.2004.2813.

2. Phillips SJ, Dudik M (2008) Modeling of species distributions with Maxent: new extensions and a comprehensive evaluation. Ecography 31: 161–175. doi:10.1111/j.0906-7590.2008.5203.x.

3. Elith J, Phillips SJ, Hastie T, Dudik M, Chee YE, et al. (2010) A statistical explanation of MaxEnt for ecologists. Diversity and Distributions 17: 43–57. doi:10.1111/j.1472-4642.2010.00725.x.

4. Hijmans R, Cameron S, Parra J, Jones P, Jarvis A (2005) Very high resolution interpolated climate surfaces for global land areas. International Journal of Climatology 25: 1965–1978.

5. Breiman L (2001) Statistical modeling: The two cultures (with comments and a rejoinder by the author). Statist Sci 16: 199–231.

6. Strobl C, Malley J, Tutz G (2009) An introduction to recursive partitioning: Rationale, application, and characteristics of classification and regression trees, bagging, and random forests. Psychological Methods 14: 323–348. doi:10.1037/a0016973.

7. Evans SN, Hower V, Pachter L (2010) Coverage statistics for sequence census methods. BMC Bioinformatics 11: 430. doi:10.1186/1471-2105-11-430.

8. Svetnik V, Liaw A, Tong C, Wang T (2004) Application of Breiman’s Random Forest to Modeling Structure-Activity Relationships of Pharmaceutical Molecules. In: Roli F, Kittler J, Windeatt T, editors. Lecture Notes in Computer Science. Berlin: Springer, Vol. 3077. pp. 334–343.

9. Larison B, Njabo KY, Chasar A, Fuller T, Harrigan RJ, et al. (2014) Spillover of pH1N1 to swine in Cameroon: an investigation of risk factors. BMC Vet Res 10: 1–8. doi:10.1186/1746-6148-10-55.
